# Prevalence of Intracranial Aneurysms in Patients With Coarctation of the Aorta: A Systematic Review and Meta-Analysis

**DOI:** 10.1016/j.jacadv.2023.100394

**Published:** 2023-07-05

**Authors:** Alvan D. Buckley, Kevin Yo Han Um, Javier I. Ganame, Omid Salehian, Arsha Karbassi

**Affiliations:** Division of Cardiology, McMaster University, Hamilton, Ontario, Canada

**Keywords:** angiography, congenital heart disease, screening

## Abstract

**Background:**

Coarctation of the aorta (CoA) is associated with intracranial aneurysms (IAs); however, the prevalence and risk factors (RFs) are not well described. Current practice guidelines offer inconsistent recommendations on screening for IAs in this patient population ranging from “not recommended” (European Society of Cardiology 2020) to “recommended” (American Heart Association 2018).

**Objectives:**

The purpose of this study was to determine the prevalence and RFs for IAs in patients with CoA.

**Methods:**

We completed a systematic review and meta-analysis of studies utilizing computed tomography or magnetic resonance angiographic screening for IAs in patients with CoA.

**Results:**

Five cohort studies were included, representing 442 patients. The pooled prevalence of IAs in patients with CoA was 3.8% [95% CI: 0.1%-12.3%]. The results met our prespecified definition for high heterogeneity. Of 5 RFs evaluated, only hypertension was associated with the development of IAs with an odds ratio of 3.1 [95% CI: 1.1-8.2; *P* = 0.03]. There was an observed downward trend over time in the prevalence of IAs among the studies included.

**Conclusions:**

The development of IAs is likely multifactorial in etiology and there may be modifiable RFs in their development. Considering the low prevalence of IAs in the pooled result, routine screening of patients with CoA for IAs is likely of low-value.

Coarctation of the aorta (CoA) is found in approximately 4 of 10,000 live births, accounting for 5% of congenital heart diseases (CHD).^1^^,^^2^ Despite relief of the anatomical obstruction the subsequent risk of early morbidity and mortality persists, with more recent studies reporting an average survival to 60 years of those who reach adolescence.^3^^,^^4^ Patients with CoA are at increased risk for hypertension (HTN), stroke, aortic dissection, coronary artery disease, and heart failure.^3^^,^^4^ Several studies have reported on the prevalence of intracranial aneurysms (IAs) in this population with earlier studies estimating a prevalence of 10% and more recent studies reporting a prevalence of 0%.^5^, ^6^, ^7^, ^8^, ^9^, ^10^, ^11^, ^12^, ^13^ In contrast, approximately 3.2% of the general population are found to have an IA.^14^^,^^15^

There are uncertainties whether the benefits of screening patients with CoA for IAs outweigh the risks. Current practice guidelines offer inconsistent recommendations. The 2020 European Society of Cardiology guidelines state that “routine screening is not recommended,” whereas the 2018 American Heart Association (AHA)/American College of Cardiology guidelines recommend screening in adults with the caveat that “timing and frequency of screening remain a knowledge gap.”^16^^,^^17^ The 2015 Guidelines for the Management of Patients With Unruptured Intracranial Aneurysms by the AHA/American Stroke Association state that it is reasonable to offer computed tomography angiography (CTA) or magnetic resonance angiography (MRA) to patients with CoA (Class IIa; Level of Evidence: B).^14^ This systematic review and meta-analysis of the current literature assesses the prevalence of IAs in patients with CoA. In addition, we analyze the association between reported risk factors (RFs) and prevalence of IAs, in an effort to further our understanding of this association and its implications for screening.

## Methods

This study was conducted according to the 2009 Preferred Reporting Items for Systematic Reviews and Meta-Analyses guidelines.^18^ Ethical approval was not required as patient-specific data were not acquired, and all data involved are publicly accessible.

### Search strategy

We carried out a systematic search of the literature in Medline, Medline In-Process, EMBASE, and Cochrane Central Register of Clinical Trials databases. A librarian aided in the development and completion of the search. Where available, both controlled vocabulary terms including Medical Subject Heading terms and keywords were used in the subject component blocks. We used the following combination of terms: “(CoA, CHD) AND (IA, subarachnoid hemorrhage, stroke) AND (computerized tomography, magnetic resonance imaging, angiography, screening, prevalence).” The search was performed for studies published up to February 2021, and updated to March 2022, and limited to humans. Non-English language studies were included and translated when necessary through translators at McMaster University. Additional studies were identified by searching article reference lists and searching the gray literature. Search results were imported into Covidence systematic review software (Veritas Health Innovation). Patients were not involved in the generation of this meta-analysis.

### Eligibility criteria

Inclusion criteria for this systematic review were the following: 1) prospective and retrospective studies reporting on the prevalence of IAs in patients with CoA and; 2) the population underwent screening using angiographic imaging using CTA or MRA as these modalities are considered to have >99% sensitivity and specificity for the detection of IAs. Exclusion criteria were the following: 1) case series or case reports as they represented a biased cohort; 2) studies reporting on patients with coarctation of the abdominal aorta; 3) studies including patients that are <1 year of age; and 4) patients with bicuspid aortic valve (BAV) but no CoA. Two independent reviewers (A.B., A.K.) assessed studies for eligibility and recorded the main reason for exclusion. Disagreements on which studies to include were resolved by discussion or a third reviewer if needed. If more than 1 publication was found presenting the same patient data, the most recent study was included.

### Data extraction and collection

Two authors reviewed the included studies and extracted relevant data. Standardized case report forms were used to record the following details where available: study metadata, eligibility criteria, patient characteristics, severity and surgical status of CoA, comorbidities, screening method, and prevalence and size of IAs found. When adequate information was not available in the publication, the corresponding authors of the studies were contacted. Results were then compared and disagreements were resolved through discussion and rereview of the articles.

### Quality assessment

Studies were evaluated for risks of biases using a modified version of the Newcastle-Ottawa Quality Assessment Scale for Cohort Studies.^19^ This scale assesses quality in several domains: sample selection, comparability, and outcomes. Total scores range from 0 to 7. Studies were judged to be at low risk of bias (≥4 points) or high risk of bias (<4 points). Two independent reviewers evaluated each study independently and discrepancies were discussed among the reviewers and settled through consensus.

### Study outcome

The primary outcome was the prevalence of IAs in patients with CoA, as detected by CTA or MRA. Secondary outcomes include site-specific distribution of IAs where available, size of IAs, and the association of cardiovascular RFs with prevalence of IAs. RFs included were: age, sex, presence of a BAV, HTN, and history of smoking.

### Statistical analysis

We completed an initial descriptive analysis of the studies. We used DataParty and R Software 4.1.1 to perform the meta-analysis of prevalence.^20^^,^^21^ We transformed the prevalence for each study using the double arcsine method.^22^ We then performed sensitivity analyses with both logit and arcsine square root transformations. We weighted the estimates by inverse variance and applied a random effects model using the DerSimonian-Laird Method. We back-transformed the pooled prevalence of IAs and their 95% CIs for ease of interpretation. We presented the degree of heterogeneity between estimates using the tau^2^ and I^2^ statistics. We considered an I^2^ value above 75% to indicate high heterogeneity.^23^ Further, we pooled comorbidities reported in 2 or more studies using a random-effects model. Dichotomous variables and continuous variables were pooled as odds ratios and standardized mean differences, and reported through forest plots.

## Results

### Search results and study selection

**Figure 1:**
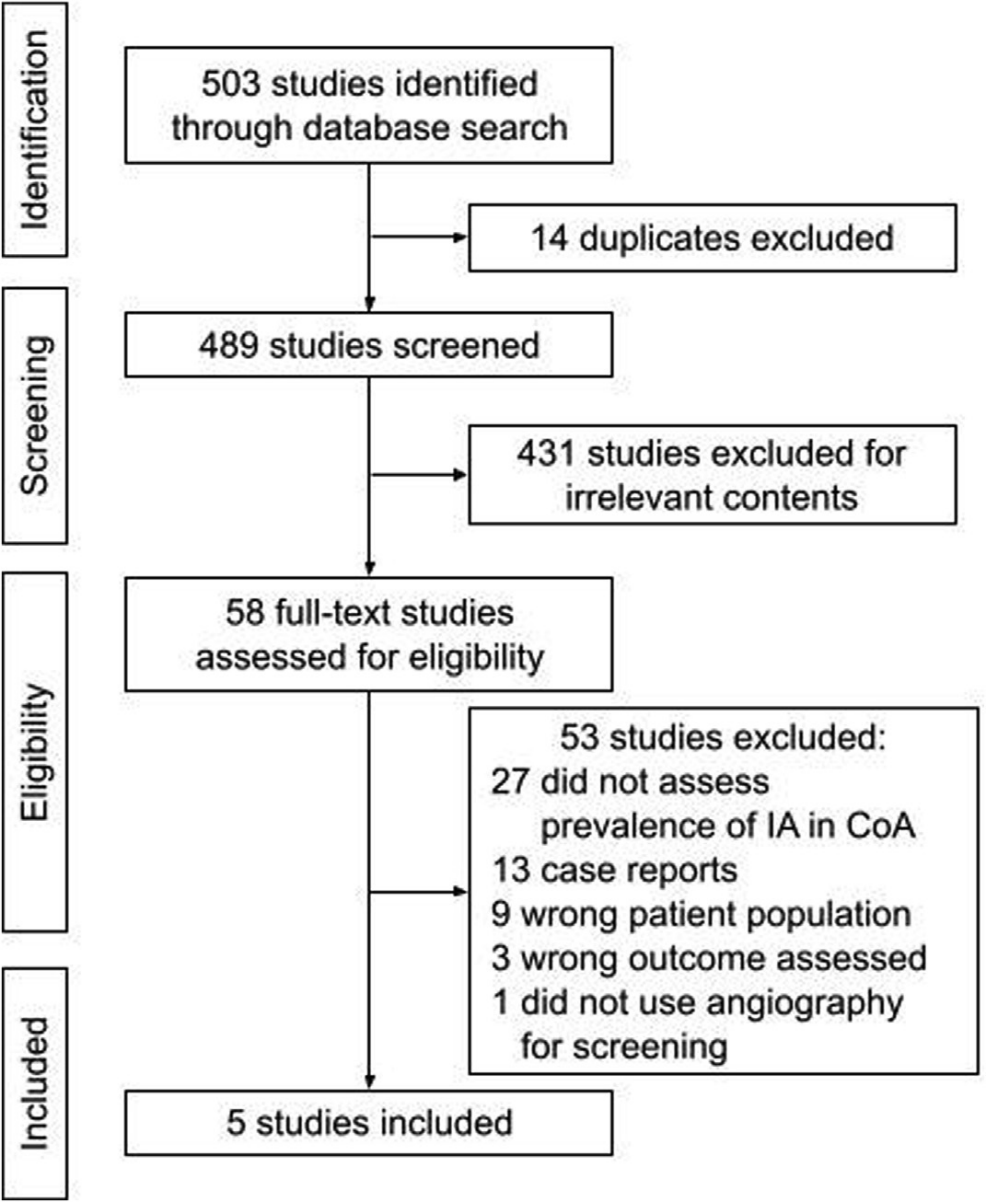
**PRISMA Diagram of Search Strategy** Preferred Reporting Items for Systematic Reviews and Meta-Analyses diagram of search strategy demonstrating number of studies identified, screened, deemed eligible, and included in the meta-analysis. CoA = coarctation of the aorta; IA = intracranial aneurysm.

We identified 489 unique citations, from which 58 full texts were assessed for eligibility. Five cohort studies met inclusion criteria for the primary outcome and were included in the meta-analysis. The most common reason studies were excluded was due to a lack of screening for IAs in patients with CoA, followed by a study design not included in this analysis (eg, case reports). Figure 1 demonstrates the Preferred Reporting Items for Systematic Reviews and Meta-Analyses diagram of the search.

### Study characteristics

Of the 5 cohort studies included, 3 were prospective and 2 were retrospective in design (Table 1). They were published between 2003 and 2020, representing patients who were imaged between the years of 1980 to 2018. The imaging modalities used to screen for IA’s included CTA and MRA. Two studies included adults ≥18 years of age, one study included individuals ≥17 years of age, another included patients ≥13 years of age, and the Donti study included patients with a mean age of 15.7 ± 7.1 years. All included studies were deemed to have a low risk of bias based on the modified Newcastle-Ottawa Quality Assessment Scale for Cohort Studies as shown in Table 2.

**Table 1:** Characteristics of Included Studies

First Author	Years Patient Referred	Ages Included, y	Patients Excluded	Screening Modality	N	Number of Patients With IAs (%)
Connolly et al^5^	1980-2002	≥18	Implanted device	MRA	100	10
Curtis et al^6^	1999-2007	≥17	None	MRA	117	12
Cook et al^7^	2008-2011	≥18	Pregnant, CKD	CTA	43	6
Donti et al^8^	2004-2011	Mean 15.7	Untreated CoA, known RFs for IA	MRA	80	0
Andrade et al^11^	2000-2018	≥13	None	CTA or MRA	102	0

CoA = coarctation of the aorta; CTA = computed tomography angiography; IA = intracranial aneurysm; MRA = magnetic resonance angiography; RF = risk factor.

**Table 2:** Modified Newcastle-Ottawa Quality Assessment Scale for Cohort Studies

First Author	Selection				Comparability	Outcome		Total Score (Max = 7)
	Sample Representativeness	Sample Size	Definition of Controls	Ascertainment of CoA	Comparability of Design or Analysis	Ascertainment of IA	Quality of Statistical Analysis	
Connolly et al^5^	0	1	1	1	0	1	1	5
Curtis et al^6^	1	1	1	1	1	1	1	7
Cook et al^7^	0	0	1	1	0	1	1	4
Donti et al^8^	1	0	1	1	1	1	1	6
Andrade et al^11^	0	1	1	1	1	1	1	6

CoA = coarctation of the aorta; IA = intracranial aneurysm.

### Prevalence of IAs

**Figure 2:**
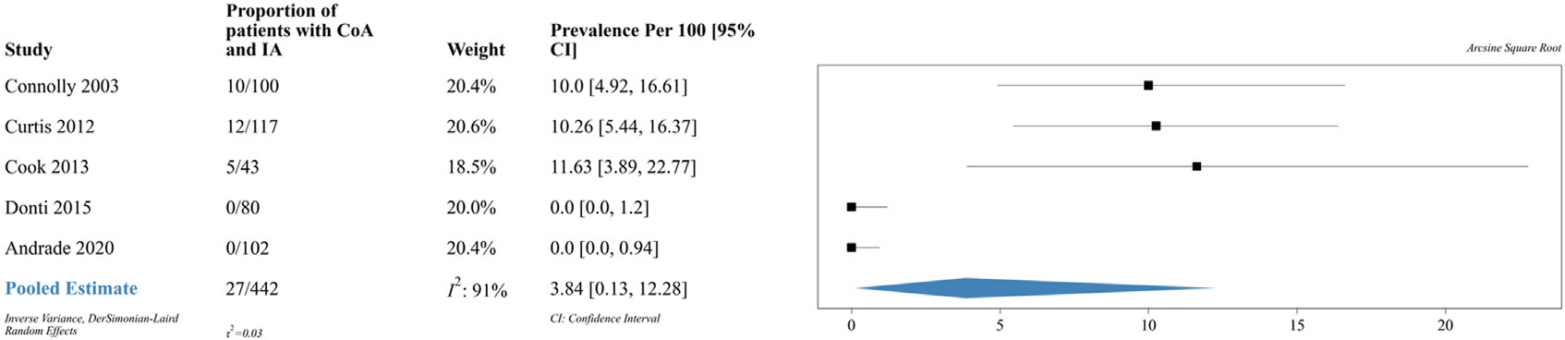
**Forest Plot of Prevalence of IA in Patients With CoA** Reported prevalence and pooled estimate of proportion of patients with coarctation of the aorta who have an intracranial aneurysm. **Black square**: point estimate of prevalence. **Blue rhombus**: pooled estimate and confidence interval of prevalence. CoA = coarctation of the aorta; IA = intracranial aneurysm.

Data on the primary outcome of interest were available for a total of 442 patients with CoA. A total of 27 (6%) patients with CoA were found to have an IA, of which 1 patient had 2 IAs. The final, pooled, prevalence of IAs was 3.8% [95% CI: 0.1%-12.3%] as shown in Figure 2. The I^2^ heterogeneity statistic was 91% and met our prespecified requirement of high heterogeneity. Sensitivity analysis with logit and arcsine square root transformations showed similar results as shown in Supplemental Figures 1 and 2.

**Figure 3:**
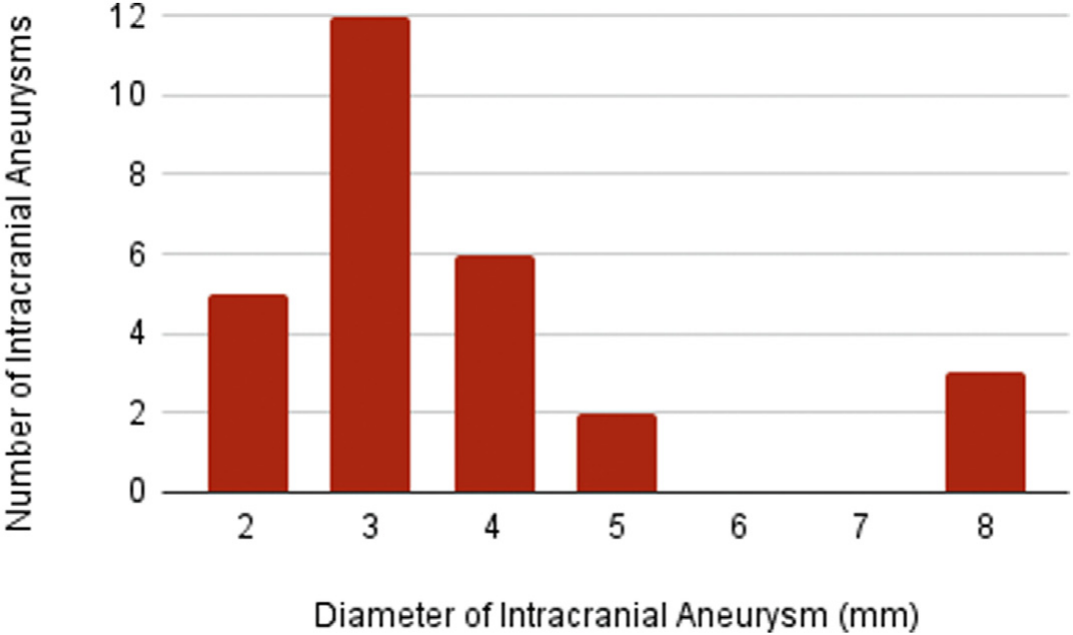
**Number of IAs** Number of intracranial aneurysms by diameter in mm (total of 28).

Characteristics of the 28 IAs found (1 patient had 2 IAs) are outlined in Figure 3 and Table 3. Of note, all 3 aneurysms that were 8 mm in size were found in the basilar tip region. Of the 27 patients found to have an IA, 6 were referred for neurosurgical assessment. Three of these 6 patients underwent intervention, with 1 suffering cognitive deficit secondary to a subarachnoid hemorrhage at the surgical site. The study by Connolly et al noted that serial imaging was being completed every 6 to 12 months to monitor the IAs found in their cohort. They reported no change in aneurysm size or number but there was no comment on duration of follow-up after initial detection of an IA. The study by Curtis et al reported that their group was carrying out annual MRAs for both patients with aneurysms 5 mm or larger, but did not comment on any changes or duration of follow-up.

**Table 3:** Characteristics of the 27 Patients Found to Have an IA

Total number of intracranial aneurysms	28
Age, y	37.5 ± 12.5
Female	9 (33.0)
Location of intracranial aneurysm	
Middle cerebral artery	8
Posterior cerebral artery	2
Anterior cerebral artery	2
Anterior communicating artery	2
Posterior communicating artery	3
Basilar tip	3
Superior cerebellar artery	2
Posterior inferior cerebellar artery	1
Internal carotid	3
Anterior choroidal artery	2

Values are n, mean ± SD, or n (%).

IA = intracranial aneurysm.

### Risk factors

Characteristics and comorbidities of the patients represented in the 5 studies are summarized in Table 4, the Central Illustration, and Supplemental Figures 3-8. The mean difference of ages between patients who had an IA compared to those who did not have an IA was 6.0 years [95% CI: −4.8 to 16.7; *P* = 0.28]. Supplemental Table 1 displays the ages of the participants across the 5 studies. The Donti and Andrade studies were excluded in this pooled estimate as there were no patients in the IA arm of either study making it impossible to include them in a pooled estimate for mean difference of ages. Odds ratios were only possible for 4 dichotomous variables: sex, presence of a BAV, HTN, and history of smoking. All other variables were either not included in enough studies, had insufficient data points, or were not compared in the studies between the 2 groups of interest. Patients with a diagnosis of HTN were found to be more likely to have an IA with an odds ratio of 3.1 [95% CI: 1.1-8.2; *P* = 0.03]. The other 3 dichotomous variables evaluated showed no statistically significant differences as shown in Table 4 and Supplemental Figures 3-7, found in Supplemental Appendix B.

**Table 4:** Characteristics of Patients Enrolled in the 5 Studies

	Connolly et al^5^ (n = 100)	Curtis et al^6^ (n = 117)	Cook et al^7^ (n = 43)	Donti et al^8^ (n = 80)	Andrade et al^11^ (n = 102)	Odds Ratios/Mean Differences
Number of IAs observed	10	12	5	0	0	-
Female	30 (30.0)	46 (39.0)	25 (58.0)	28 (35.0)	48 (47.0)	0.8 [95% CI: 0.3-1.8; *P* = 0.6-]
Age at MRA/CTA, y	41.6 ± 16.5	27 ± 11	29 (16-59)	15.7 ± 7.1	28.9 (14.7-73.2)	6.0 [95% CI: −4.8 to 16.7; *P* = 0.28]
Age at CoA diagnosis, y	17.3 ± 16.3	-	-	2.3 ± 4.2	68 d (0-52.14 y)	-
BAV	75 (77.0)	47 (40.0)	32 (74.0)	38 (47.5)	67 (66.0)	1.0 [95% CI: 0.5-2.3; *P* = 1.0-]
History of CoA repair	86 (86.0)	107 (91.0)	43 (100.0)	80 (100.0)	99 (97.0)	-
Age at diagnosis of CoA, y	17.3 ± 16.3	-	-		68 d (0-52 y)	-
Age at first CoA repair, y	-	-	9.8 ± 11.3	2.6 ± 4.4	4.4 mo (2 d-47 y)	-
Treated for recoarctation	19 (19%)	-	13 (30.0)	13 (16.0)	35 (34.0)	-
VSD				22 (28.0)	(27.5)	-
Neurologic symptoms	35 (36.0)	-	-	9 (11.2)	(60.0)	-
Hypertension	63 (63.0)	53 (45.0)	-	6 (7.5)	13 (13.0)	3.1 [95% CI: 1.1-8.2; *P* = 0.03]
History of smoking	41 (43.0)	19 (17.0)	4 (9.0)	0	13 (13.0)	2.0 [95% CI: 0.8-5.0; *P* = 0.12]
Personal history of IA	1 (1.0)	-	-	0	0	-
Family history of IA	3 (5.0)	-	-	0	2 (2.0)	-
Diabetes	9 (9.0)	-	-	-	4 (4.0)	-

Values are n, n (%), mean ± SD, or median (range) unless otherwise indicated.

BAV = bicuspid aortic valve; CoA = coarctation of the aorta; CTA = computed tomography angiography; IA = intracranial aneurysm; MRA = magnetic resonance angiography; VSD = ventricular septal defect.

**Central Illustration:**
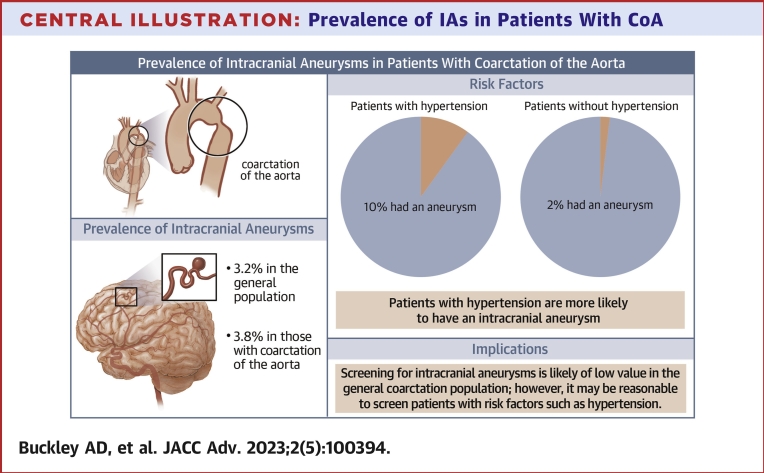
**Prevalence of IAs in Patients With CoA** This illustration depicts the reported prevalence of intracranial aneurysms in the general population compared to the pooled result of our meta-analysis, along with risk factors associated with their development in this patient population. Hypertension was statistically significantly associated with the development of intracranial aneurysms. Implications of these results are pointed out. CoA = coarctation of the aorta.

## Discussion

The meta-analysis performed for this study resulted in an estimated prevalence of IAs in patients with CoA to be approximately 3.8% [95% CI: 0.1%-12.3%]. This is compared to a reported prevalence of 3.2% in the general population.^14^ However, there was significant statistical heterogeneity in the results of the 5 studies included in the meta-analysis, thus the appropriateness of calculating a pooled prevalence may be questioned. Considering the similar methodology across the studies, including the use of high fidelity angiographic modalities, the high statistical heterogeneity is likely due to heterogeneity of the populations between studies.

Although the pooled prevalence suffered from high heterogeneity, it is notable that the reported prevalence of IAs among individual studies showed a marked downward trend toward the more recent studies. There are many potential explanations for this observed trend, including random chance. Importantly, the oldest study represented patients who were born in the 1950s to 1990s, with each subsequent study representing patients born in more recent years. The average age at diagnosis of CoA in the Connolly study was 17 years, and in the Andrade study the age was 68 days. This marked difference was associated with earlier ages at time of intervention: >17 years vs within the first 6 months of life for the Connolly and Andrade studies, respectively. It is possible that earlier detection and earlier intervention with modern techniques for CoA reduces potential long-term complications associated with CoA, such as the development of IAs. Perhaps modern day treatment of HTN and diabetes is implemented at an earlier age, or more effectively, affecting the observed prevalence of IAs. Similarly, there has been a decrease in the prevalence of smoking in the general population which may have translated to fewer patients developing IAs. Further, the organization and implementation of adult CHD services across the globe may be better suited at following and managing this population cohort. It is also possible that, with more advanced imaging techniques and more frequent screening at a young age, we are detecting more cases of CoA that are of less severity than previous generations, which may translate to a lower prevalence of IAs. This underlines the importance of treating known modifiable RFs including smoking cessation, and HTN. These observations reflect the evolving management of patients with CHD and early vs modern surgical eras.

Nongenetic RFs for the development and progression of IAs have been documented among the general population. These include being female (thought to be due to the estrogen deficiency of menopause), smoking, and HTN. Our study found that HTN had a statistically significant association with the development of IAs in patients with CoA. This highlights the importance of RF modification in patients with CoA. In the current era there is wide screening with effective options in the management of HTN—this may have influenced the lower prevalence of IAs in the Donti and Andrade studies. Of note, females were not found to have a statistically significant association with the development of IAs in this study (odds ratio: 0.8 [95% CI: 0.3-1.8; *P* = 0.60]); however, there is a signal for lower rates of IAs in females. This may be a reflection of the age of females included in this study as the majority were premenopausal. These confounders suggest that it is not simply the presence of CoA at birth, or the associated arteriopathy, that is the single predictor of IAs in this population. Rather, the downward trend suggests that there is a modifiable RF that has reduced the frequency at which IAs develop.

The 2015 AHA/American Stroke Association guidelines for the management of patients with unruptured IAs classify IAs as being small if they are <7 mm in diameter.^14^ This group was identified as having an “extremely low risk of rupture.” Of the 28 IAs detected in the screening studies included in this analysis, 25 were <7 mm in diameter while the remaining 3 were reported to be 8 mm in diameter. Thus, only 3 patients out of 442 were found to have an IA warranting a patient-physician discussion about the benefits and risks of intervention. It is unclear if patients with smaller IAs warrant serial imaging to monitor for progression in size or number of IAs. If the 7 mm diameter cut-off is used to define a clinically relevant IA, then there was a very low rate (0.7%) of clinically relevant IAs found in this population.

A recent decision and cost-effectiveness analysis was carried out by Pickard et al^24^ to determine appropriate age and interval of screening for IAs in patients with CoA. They concluded that there is a benefit to screening at ages 10 and 20 years of age. The study was, however, limited in that their estimated prevalence of IAs in this population was 12% which, based on our results, may reflect an older generation. The clinical significance of smaller IAs was not taken into account, and it was assumed that small IAs were expected to grow which conflicts with the 2015 AHA/ASA guidelines. They also assumed that the rate of IA development to be constant throughout life (0.439% per year). It is not clear that the rate of IA development is constant throughout life—there may be a peak age at which IAs develop, particularly in females around the age of menopause. Prevalence studies in the general population show an increase in frequency of IAs by age with a peak in the fifth and sixth decade of life.^14^ Finally, their recommendation to screen patients at 10 and 20 years conflicts with the results from 2015 Donti study which failed to find any IAs in 80 patients with a mean age of 16.

Considering the drive for value-based care,^25^ reducing low-value imaging may aid in containing health care costs and preventing risks associated with overuse. The evidence for imaging patients with CoA for IAs remains uncertain, and based on the results of this review routine screening is likely of low-value and economically inefficient. A nuanced approach taking into account a patient's symptoms and risk profile (eg, age, HTN) may be more appropriate in guiding the use of imaging to screen for IAs.

### Study Limitations

Our study is limited in that there was a small number of eligible studies included in the meta-analysis. This likely contributed to the significant heterogeneity observed. Overall, only 442 patients were represented by these 5 studies. As a result, there was insufficient data to calculate age- and sex-specific prevalences of IAs in patients with CoA. Notably, the earlier studies included patients born in the 1950s when diagnostic modalities and interventions for CoA were significantly less advanced than what we have available today—thus, the observations from these older cohorts may not be as valid for current patients. Two of the studies included were retrospective in design, introducing the potential for referral bias which may have led to increased prevalence. This is highlighted by the observation that 36% of patients in the Connolly study reported neurological symptoms. A significant assumption of this meta-analysis was that the age distribution of the patients included followed a binomial distribution—this may have negatively impacted the standardized mean difference of ages.

## Conclusions

Our results reveal a pooled estimated prevalence of IAs in patients with CoA to be approximately 3.8%, which is marginally higher than that reported in the general population (3.2%). However, the meta-analysis did suffer from significant statistical heterogeneity. There was an observed downward trend over time in the prevalence of IAs among the individual studies included which is likely multifactorial in etiology and suggests that there are modifiable RFs in the development of IAs in this population. Routine screening of asymptomatic patients with CoA for IA is likely of low-value. However, it may be reasonable to screen patients with associated risks including older age, and HTN. This area of study would benefit from further prospective studies evaluating the prevalence of IAs in adults with CoA to further the discussion on whether these patients should be screened for IAs.

## Funding support and author disclosures

The authors have reported that they have no relationships relevant to the contents of this paper to disclose.

PERSPECTIVES **COMPETENCY IN MEDICAL KNOWLEDGE:** CoA carries lifelong mortality and morbidity implications despite early intervention. The prevalence of IAs in this population has decreased over time and may be similar to the general population in the modern era. Based on currently available literature, universal screening of patients with CoA with angiographic imaging is likely of low value.

**TRANSLATIONAL OUTLOOK:** Further studies are warranted to understand the modern prevalence of and RFs associated with IAs in patients with CoA.
