# Supplementary material for: Prevalence of Intracranial Aneurysms in Patients With Coarctation of the Aorta: A Systematic Review and Meta-Analysis
Source: JACC Adv. 2023 Jul 5;2(5):100394. doi: 10.1016/j.jacadv.2023.100394 (PMC11198084; doi:10.1016/j.jacadv.2023.100394)
Supplement: Supplemental Figures 1-8 and Table 1 [file mmc1.pdf]

SUPPLEMENTAL APPENDICES

Appendix A: Ages of the participants across the five studies

The average age of participants included in each study are displayed in Supplemental Table 1. The age of participants with and without an intracranial aneurysm are also displayed.

Appendix B: Sensitivity analysis of primary outcome

The final, pooled, prevalence of IAs was 3.8% (95% CI, 0.1% to 12.3%) as shown in Figure 2 using an arcsine square root transformation. As part of the sensitivity analysis, similar analyses were undertaken with logit and Freeman Tukey Double Arcsine transformations as shown in Supplemental Figures 1 and 2. These analyses shower similar results in terms of the wide variance in the pooled estimate. The logit transformation suffers as the studies with zero cases of IA's are excluded from the pooled result.

Appendix C: Forest plots of risk factors evaluated

In the Donti study all 80 participants were non-smokers, thus the denominator in the comparator arm (smokers) was zero meaning the meta-analysis of this risk factor was not possible. As per convention we performed a sensitivity analysis whereby the Donti study was not included, and a second analysis where 0.5 and 1 were added to the numerator and denominator, respectively, of both the comparator and control arms of this study. The results of these two meta-analyses were similar with statistically non-significant p values, as shown in Supplemental Figures 6 and 7.

In the Donti and Andrade studies there were no patients in the comparator arm (participants with IA's). Thus, there were no participants in the denominator of the comparator arm. As per convention these studies were excluded from the pooled estimate - this introduces significant bias as these two studies clearly found lower rates of IA's in their cohorts which included younger patients. We have included the pooled estimate of the other three studies in Supplemental Figure 8.

SUPPLEMENTAL FIGURES

Supplemental Figure 1: Forest plot using Freeman Tukey Double Arcsine transformation

Study	Patients with CoA and IA	Weight	Prevalence Per 100 [95% CI]
Connolly 2003	10/100	20.5%	10.0 [4.78, 16.75]
Curtis 2012	12/117	20.8%	10.26 [5.33, 16.48]
Cook 2013	5/43	18.1%	11.63 [3.46, 23.2]
Donti 2015	0/80	20.0%	0.0 [0.0, 2.14]
Andrade 2020	0/102	20.6%	0.0 [0.0, 1.68]
**Pooled Estimate**	27/442	I^2: 88%	4.79 [0.60, 12.45]

Inverse Variance, DerSimonian-Laird Random Effects

τ^2=0.02

CI: Confidence Interval

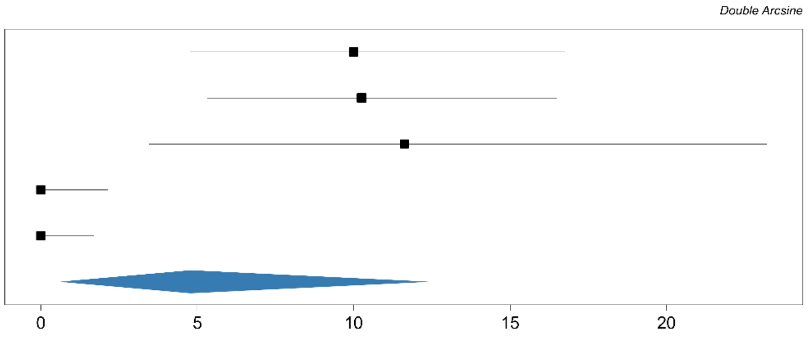

Caption: Reported prevalence and pooled estimate of proportion of patients with coarctation of the aorta who have an intracranial aneurysm using a Freeman Tukey Double Arcsine transformation.

CoA, coarctation of the aorta; IA, intracranial aneurysm.

Supplemental Figure 2: Forest plot using Logit transformation

Study	Patients with CoA and IA	Weight	Prevalence Per 100 [95% CI]
Connolly 2003	10/100	37.2%	10.0 [5.47, 17.6]
Curtis 2012	12/117	44.5%	10.26 [5.92, 17.2]
Cook 2013	5/43	18.3%	11.63 [4.92, 25.05]
**Pooled Estimate**	27/260	I^2: 0%	10.40 [7.23, 14.74]

Inverse Variance, DerSimonian-Laird Random Effects τ^2=0.00 *CI: Confidence Interval*

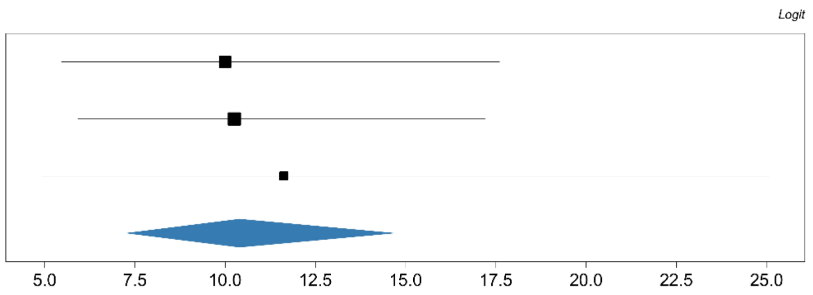

Caption: Reported prevalence and pooled estimate of proportion of patients with coarctation of the aorta who have an intracranial aneurysm using a Logit transformation.

CoA, coarctation of the aorta; IA, intracranial aneurysm.

Supplemental Figure 3: Forest plot comparing prevalence in females versus males

IA in CoA - Risk Factors (F vs M)

Study	Female	(%)	Male	(%)	Weight	OR [95% CI]
Connolly 2003	2/30	(7%)	8/70	(11%)	27.1%	0.55 [0.11, 2.78]
Curtis 2012	4/46	(9%)	8/71	(11%)	44.3%	0.75 [0.21, 2.65]
Cook 2013	3/25	(12%)	2/18	(11%)	19.5%	1.09 [0.16, 7.31]
Donti 2015	0/28	(0%)	0/52	(0%)	4.5%	1.84 [0.04, 95.33]
Andrade 2020	0/48	(0%)	0/54	(0%)	4.5%	1.12 [0.02, 57.72]
**Pooled Estimate**	9/177	(5%)	18/265	(7%)	I^2: 0%	0.79 [0.34, 1.83]
Mantel-Haenszel, DerSimonian-Laird Random Effects	*p=0.58, z=0.56 τ^2=0.00*					*OR: Odds Ratio CI: Confidence Interval*

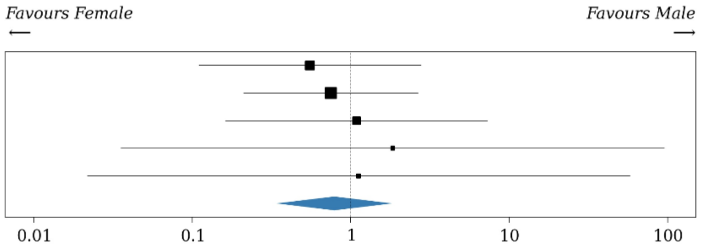

Caption: Reported frequency and pooled odds ratio for females and risk of having an IA.

CoA, coarctation of the aorta; IA, intracranial aneurysm.

Supplemental Figure 4: Forest plot comparing prevalence in patients with BAV versus TAV

IA in CoA - Risk Factors (BAV vs TAV)

Study	Bicuspid AV	(%)	Tricuspid AV	(%)	Weight	OR [95% CI]
Connolly 2003	3/25	(12%)	7/75	(9%)	32.7%	1.32 [0.32, 5.57]
Curtis 2012	7/70	(10%)	5/47	(11%)	45.9%	0.93 [0.28, 3.14]
Cook 2013	1/11	(9%)	4/32	(12%)	12.7%	0.7 [0.07, 7.03]
Donti 2015	0/52	(0%)	0/28	(0%)	4.3%	0.54 [0.01, 28.09]
Andrade 2020	0/35	(0%)	0/67	(0%)	4.3%	1.9 [0.04, 97.87]
**Pooled Estimate**	11/193	(6%)	16/249	(6%)	I^2: 0%	1.02 [0.45, 2.31]
Mantel-Haenszel, DerSimonian-Laird Random Effects	*p=0.97, z=0.04 τ^2=0.00*					*OR: Odds Ratio CI: Confidence Interval*

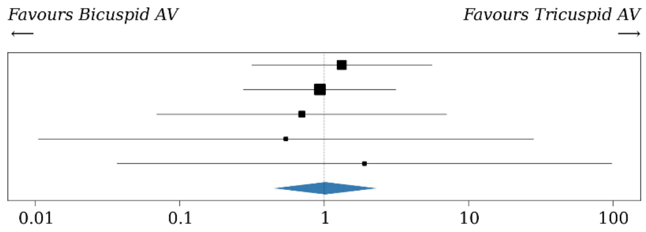

Caption: Reported frequency and pooled odds ratio for participants with a bicuspid aortic valve and risk of having an IA.

CoA, coarctation of the aorta; IA, intracranial aneurysm.

Supplemental Figure 5: Forest plot comparing prevalence of IAs in patients with HTN

IA in CoA - Risk Factors (HTN vs Normotensive)

Study	Hypertension	(%)	Normotensive	(%)	Weight	OR [95% CI]
Connolly 2003	7/63	(11%)	3/37	(8%)	48.2%	1.42 [0.34, 5.85]
Curtis 2012	10/53	(19%)	2/64	(3%)	39.5%	7.21 [1.5, 34.56]
Donti 2015	0/6	(0%)	0/74	(0%)	6.1%	11.46 [0.21, 626.48]
Andrade 2020	0/41	(0%)	0/61	(0%)	6.2%	1.48 [0.03, 76.18]
**Pooled Estimate**	17/163	(10%)	5/236	(2%)	I^2: 0%	3.07 [1.14, 8.21]
Mantel-Haenszel, DerSimonian-Laird Random Effects	*p=0.03, z=2.23* τ^2*=0.00*					*OR: Odds Ratio CI: Confidence Interval*

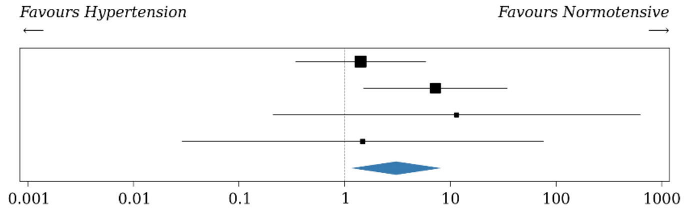

Caption: Reported frequency and pooled odds ratio for participants with hypertension and risk of having an IA.

CoA, coarctation of the aorta; IA, intracranial aneurysm.

Supplemental Figure 6: Forest plot comparing prevalence of IAs in patients who smoke

IA in CoA - Risk Factors (Smoker vs Non-smoker)

Study	Smoker	(%)	Non-smoker	(%)	Weight	OR [95% CI]
Connolly 2003	6/41	(15%)	4/59	(7%)	45.5%	2.36 [0.62, 8.95]
Curtis 2012	3/19	(16%)	9/98	(9%)	40.7%	1.85 [0.45, 7.6]
Cook 2013	0/4	(0%)	5/39	(13%)	8.7%	0.7 [0.03, 14.82]
Andrade 2020	0/13	(0%)	0/89	(0%)	5.2%	6.63 [0.13, 348.31]
**Pooled Estimate**	9/77	(12%)	18/285	(6%)	I^2: 0%	2.03 [0.83, 4.99]

Mantel-Haenszel, DerSimonian-Laird Random Effects *$p=0.12$, $z=1.54$ $\tau^2=0.00$* *OR: Odds Ratio CI: Confidence Interval*

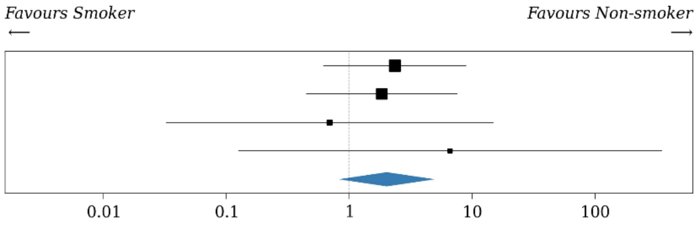

Caption: Reported frequency and pooled odds ratio for participants with a history of smoking and risk of having an IA with the Donti study excluded.

CoA, coarctation of the aorta; IA, intracranial aneurysm.

Supplemental Figure 7: Forest plot comparing prevalence of IAs in patients who smoke

IA in CoA - Risk Factors (Smoker vs Non-smoker)

Study	Smoker	(%)	Non-smoker	(%)	Weight	OR [95% CI]
Connolly 2003	6/41	(15%)	4/59	(7%)	43.9%	2.36 [0.62, 8.95]
Curtis 2012	3/19	(16%)	9/98	(9%)	39.3%	1.85 [0.45, 7.6]
Cook 2013	0/4	(0%)	5/39	(13%)	8.4%	0.7 [0.03, 14.82]
Andrade 2020	0/13	(0%)	0/89	(0%)	5.0%	6.63 [0.13, 348.31]
Donti 2015	0/1	(0%)	0/81	(0%)	3.4%	161.0 [1.32, 19680.27]
**Pooled Estimate**	9/78	(12%)	18/366	(5%)	I^2: 0%	2.35 [0.97, 5.71]
Mantel-Haenszel, DerSimonian-Laird Random Effects	*p=0.06, z=1.89* $\tau^2=0.00$					*OR: Odds Ratio CI: Confidence Interval*

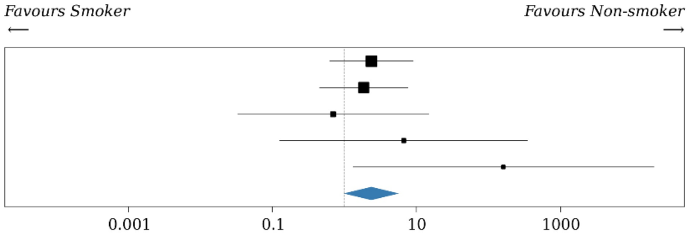

Caption: Reported frequency and pooled odds ratio for participants with a history of smoking and risk of having an IA with the Donti study included (0.5 was added to the numerator and 1 added to the denominator for both the comparator and control arms).

CoA, coarctation of the aorta; IA, intracranial aneurysm.

Supplemental Figure 8: Forest plot comparing prevalence of IAs in patients by age

IA in CoA - Risk Factors (Age)

Study	With IA	(N)	Without IA	(N)	Weight	MD [95% CI]
Connolly 2003	38 ± 10	(10)	42 ± 17	(90)	33.5%	-4 [-11.12, 3.12]
Curtis 2012	34 ± 12	(12)	27 ± 11	(105)	33.5%	7 [-0.11, 14.11]
Cook 2013	46 ± 8	(5)	31 ± 8	(38)	33.0%	15 [7.54, 22.46]
**Pooled Estimate**					I^2: 85%	5.96 [-4.77, 16.69]

Inverse Variance, DerSimonian-Laird Random Effects; *$p=0.28$, $z=1.09$, $\tau^2=76.32$*; *MD: Mean Difference, CI: Confidence Interval*

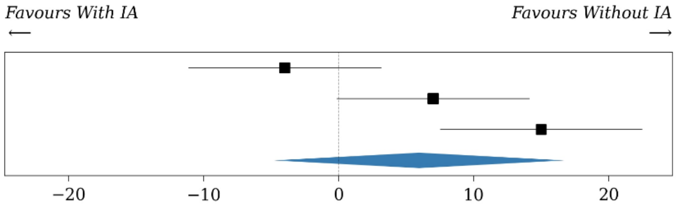

Caption: Reported mean and standard deviation of ages for participants with and without IA's in each study.

CoA, coarctation of the aorta; IA, intracranial aneurysm.

SUPPLEMENTAL TABLES

Supplemental Table 1: Comparison of ages across the five studies

Characteristic	Connolly (2003)	Curtis (2012)	Cook (2013)	Donti (2015)	Andrade (2020)
Patients	100	117	43	80	102
# of IAs Observed	10	12	5	0	0
Age at MRA/CTA	41.6 ± 16.5	29 (16-59)	33.55 ± 10.21	15.7 ± 7.1	28.9 (14.7-73.2)
Age of patients with IA	38.4 ± 10.2	37 (16-50) 33.8 ± 11.8	45.60 ± 8.17	-	-
Age of patients without IA	41.9 ± 17.1	23 (16-59) 27 ± 11	30.89 ± 7.89	15.7 ± 7.1	28.9 (14.7-73.2)

CTA, computed tomography angiography; IA, intracranial aneurysm; MRA, magnetic resonance angiography.
